# Validation of the volumetric flow cytometry for bovine sperm concentration

**DOI:** 10.1038/s41598-023-43678-7

**Published:** 2023-10-02

**Authors:** Alessia Gloria, Claudia Bracco, Emiliana Antenucci, Alberto Contri

**Affiliations:** 1 Department of Veterinary Medicine, University of Teramo, Località Piano D’Accio, 64100 Teramo, Italy; 2 Provincial Breeders Federation of Trento, Via delle Bettine 40, 38121 Trento, Italy

**Keywords:** Biological techniques, Physiology

## Abstract

Sperm concentration is a stronghold of the andrological evaluation and the production of insemination doses. The use of haemocytometers, although considered the gold standard, is difficult to apply in field conditions because it is subjective and time-consuming. The present study was designed to validate the volumetric flow cytometry (volFC) in order to estimate bovine sperm concentration, comparing it with the performances of haemocytometer, NucleoCounter, and flow cytometry with the use of fluorospheres. Compared with other methods, volFC appeared less affected by large dilution of the sample, with similar concentrations calculated in the range of dilution 1:200–1:800. Using volFc the population detected on the basis of morphological criteria and fluorescence of DNA better represents the real concentration of sperm in the sample. The volFC showed high repeatability compared with the haemocytometer (coefficient of variation 1.85% and 4.52%, respectively) and stable performances with cryopreserved samples, with negligible effects of the medium components. The present study showed that volFC is as accurate and precise as other techniques to estimate sperm concentration in bovine fresh and frozen semen, but it is less affected by operative conditions, such as sample dilution. The possibility to quantify sperm functional subpopulations by volFC could potentially implement the study of the relationship between sperm attributes and fertility.

## Introduction

Sperm concentration, i.e. the number of sperm per unit of volume, is a pillar of andrology. Although it is not considered a direct estimator of the male function^1^, concentration was effectively used to estimate the total sperm in the ejaculate. Nevertheless, the total sperm in the ejaculate, in specific conditions, could be used to estimate testicular function since it is correlated with the efficiency of spermatogenesis^2^ and therefore it represents the function of the male gonad. The total number of sperm in the ejaculate could have clinical relevance since its reduction suggests testicular dysfunction, such as hypoplasia and degeneration^3^, tumours, infections, and genetic diseases involving the pituitary gland^4^ and/or hypothalamus^5^. Thus sperm concentration represents a fundamental point of andrology clinical evaluation. Moreover, concentration is a crucial step in the handling of semen for use or preservation. In bovine artificial insemination centres, the dilution rate of the semen with specific extenders is calculated to reach desired concentration of the insemination dose from the concentration of the raw semen. Thus the more adequate the concentration estimation, the higher the optimization of insemination doses and, in turn, of the male^6,7^.

Nowadays, the gold standard to estimate sperm concentration is the counting chamber^8^. Counting chambers showed, however, wide intra- and inter-variability, together with long time to be performed^9–11^. Thus, the counting chamber could be unenforceable in those fields in which a large number of ejaculates should be processed in reduced time, such as artificial insemination centres. For this reason, several techniques were described to accelerate the procedure and improve the precision and accuracy of sperm concentration estimation in humans and domestic animals. Recently, NucleoCounter has become popular among the automated procedures to estimate sperm concentration because of its reliability and rapidity^12–14^, without possible interference of particles or media^15^. Sperm DNA, after permeabilization with a specific property diluent and loaded in a disposable cassette, is stained with a fluorescent dye (propidium iodide), making the sperm head detectable by the instrument^15^. This device is designed specifically for sperm concentration, but using a modified procedure the number of spermatozoa with membrane integrity could be also estimated: a part of the sample is permeabilized following the standard procedure, a second part is stained without permeabilization. Both parts are analysed by the instrument that calculate, by difference, the concentration of sperm with membrane integrity^16^. This procedure, however, is more expensive, due to two measurements for one sample, and requires more time to be performed; furthermore, it is limited exclusively to membrane integrity.

Flow cytometry was proposed as a precise device for automatic sperm concentration, but the inability to quantify the sample volume made this procedure not obvious. To solve this problem, several researchers introduced fluorescent beads at known concentrations into the sample. Thus, the concentration of the raw sample could be indirectly calculated by the proportion of sperm count compared with the count of fluorescent beads at known concentration^10,12,17,18^. The implementation of modern flow cytometers with a precise and settable volume of analysis has allowed the absolute count of events in the sample. Recently, flow cytometric absolute counting of human lymphocytes has been validated^19^, suggesting that this technology could be effectively introduced also to quantify cell subpopulations. Nowadays, to the best of the authors’ knowledge, limited attention has been devoted to apply flow cytometer for absolute counting of spermatozoa^20^, and no studies in bovine has been reported yet. Furthermore, the possibility to accurately quantify the total number of spermatozoa in a sample could be transferred to all the different subpopulations detected by flow cytometry during multiparametric analysis, making this technology a potent tool to assess the properties of the semen or insemination dose.

The present study was designed to validate volumetric flow cytometry for bovine sperm concentration estimation. The effect of progressive dilution on the accuracy of sperm concentration estimation using volFC and the precision, at different dilutions, of the volFC were compared with the results obtained with the NucleoCounter, considered the highest repeatable automated technique for sperm concentration^12–14^. The agreement between sperm concentration obtained using volFC and haemocytometer (Hem), NucleoCounter (NC), and flow cytometry with fluorescent bead (spFC)s was estimated. Finally, the effectiveness of volFC to estimate cryopreserved semen concentration was also tested, using the NC as a gold standard.

## Results

In the present study the data were reported as mean ± standard deviation (s.d.)

### Preliminary trial

The precision of the volume sampled by the flow cytometer was tested using fluorospheres at certified concentrations. This standard was chosen because it is used to calculate sperm concentration in non-volumetric flow cytometers^10,12,17,18^. Both the concentrations calculated on the morphological (total events) and in the fluorescent population (events after excitation at 488 nm) were consistent at every dilution tested, between 1:0 and 1:16 (*P* > 0.05). Furthermore, a relevant proximity was recorded between the concentrations calculated on beadsT and beads-488 (1,035,858.3 ± 50,951.6 and 1,027,725 ± 54,486.6, respectively) and the certified concentration of the beads (1,014,000), with a mean proximity index of 98.3 ± 0.74%, 99.08 ± 1.41%, 99.38 ± 0.43%, 98.78 ± 1.07%, and 101.48 ± 6.97% for 1:0, 1:1, 1:2, 1:4, 1:8, respectively. Only dilution 1:16 showed significantly larger concentrations (111.1 ± 3.6%; *P* = 0.004). Using the standard beads, the repeatability of the events measured without dilution and at dilution 1:4 was excellent, with a global ICC of 0.9997 for single measures. No differences were found between beads concentration in samples analysed using a worn-out peristaltic pump tube compared with a new peristaltic pump tube in both undiluted and diluted (1:4 v:v) bead samples (*P* > 0.05).

### Trial 1. Effect of dilution on concentration estimated with a volumetric flow cytometer and NucleoCounter

Sample dilution rate represents the principal limiting factor in sperm concentration analysis with volFC and NC. Volumetric FC showed almost identical values for concentration after dilution at 1:200, 1:400, and 1:800 for all the sperm populations recorded (*P* > 0.05), although no significant differences were also detected at 1:100 (*P* > 0.05). Significant differences were found at lower dilutions, between 1:12.5 and 1:25 (*P* = 0.822), and between 1:25 and 1:50 (*P* = 0.062), suggesting no reliable estimation of sperm concentration at such dilution rates. This trend was detected for evT, evM, evH+, and evM/H+.

Similarly, a low dilution rate of the sample resulted in the inability of the NucleoCounter to estimate the concentration, with a prevalence of the error message in 100% (12 out of 12) of samples diluted at 1:12.5, 100% (12 out of 12) at 1:25, 66.7% (8 out of 12) at 1:50, 8.3% (1 out of 12) at 1:100. Differently from what showed using the volFC, where the concentration at 1:200, 1:400, and 1:800 almost overlapped, a progressive and significant increase of the concentration detected by the NC was recorded between the range 1:50 and 1:100 compared with the larger dilutions (*P* = 0.815), and between 1:100 and 1:400 (*P* = 0.006) and 1:800 (*P* = 0.000). Likewise to volFC, similar values were detected between 1:200 and 1:800 (*P* > 0.05). Data recorded to detect the effect of the dilution in the estimation of sperm concentration with volFC and NC are summarized in Table 1.

**Table 1:** Concentrations (mean and standard deviation – SD) of the different subpopulations estimated with the flow cytometry and the NudeoCounter at different dilution rates (1:12.5; 1:25; 1:50; 1:100; 1:200; 1:400; 1:800).

Dilution rate	CevT	CevM	CevH +	CevM/H +	NucleoCounter
	Mean ± SD	Mean ± SD	Mean ± SD	Mean ± SD	Mean ± SD
**1:12.5**	128.3 ± 83.2^a^	114.5 ± 74.6^a^	121.5 ± 80.9^a^	108.7 ± 73.6^a^	ERROR
**1:25**	456.2 ± 119.4^ab^	413.3 ± 112.1^ab^	421.4 ± 99^ab^	383.4 ± 95.6^ab^	ERROR
**1:50**	790.5 ± 233.9^bc^	708.8 ± 230.3^ab^	694.7 ± 302.2^ab^	684.4 ± 230.2^ab^	285.1 ± 38.6^a^
**1:100**	1082.7 ± 405.4^bc^	973.6 ± 384.8^bc^	1022.4 ± 397.6^bc^	941.3 ± 382.4^bc^	650.8 ± 195.8^ab^
**1:200**	1415.3 ± 558.2^ cd^	1272.4 ± 515.8^bc^	1351.5 ± 551.6^c^	1238.8 ± 514.4^c^	1193 ± 434.7^bc^
**1:400**	1533.7 ± 636.2^d^	1384.1 ± 570.8^c^	1454.1 ± 599.5^c^	1342.1 ± 567.5^c^	1506.2 ± 598^c^
**1:800**	1505.3 ± 609.8^d^	1385.2 ± 571.3^c^	1453 ± 603^c^	1326.2 ± 548.8^c^	1721.4 ± 687.5^c^

Total events estimated with volumetric flow cytometry—CevT; events gated on sperm morphological criteria—CevM; events positive to Hoechst 33,342—CevH + ; events gated on both sperm morphology and Hoechst positivity—CevM/H +). In the same column, values with different superscripts (a/d) differ significantly (*P* < 0.05).

### Trial 2. The repeatability of volumetric flow cytometry compared with NucleoCounter to estimate sperm concentration

The repeatability of volFC in pooled samples, estimated using the ICC, was excellent for CevM (0.9993 for single measures) and CevM/H+ (0.9995 for single measures). Moderate repeatability was recorded for CevT (0.7303 for single measures) and CevH+ (0.8850 for single measures). The ICC value for NC confirmed the excellent repeatability of such a technology, with an ICC of 0.9955 for single measures.

### Trial 3. Comparison of different methods to estimate the concentration

In this study, four different methods were used to estimate sperm concentration in fresh bovine semen. The haemocytometer, used as the gold standard to measure sperm concentration, showed moderate variability between repeated analyses of the same ejaculate, with a mean CV of 4.53%.

All the automated methods showed similar variability, lesser than the Hem, with values for the CV of 1.8%, 2.1%, and 1.94% for NC, spFC, and total events volFC, respectively. Among the populations considered in the volFC, the values of the CV were 1.94% for total events, 1.97% for morphological events, 1.25% for Hoechst positive events, and 1.85% for morphological and Hoechst positive events.

According to the results of trial 1, differences were found in the different populations recorded with the volFC. The total events showed a larger concentration, but only part of this population showed DNA content, detected using the Hoechst stain (average 93.7 ± 2.9% of the total events). Similarly, part of the total event population showed FSC and SSC consistent with bovine spermatozoa (average 87.7 ± 3.8% of the total events). Thus, the subpopulation gated for sperm morphology and Hoechst positivity was the lesser subpopulation in the sample (average 81.8 ± 5.1% of the total events).

**Figure 1:**
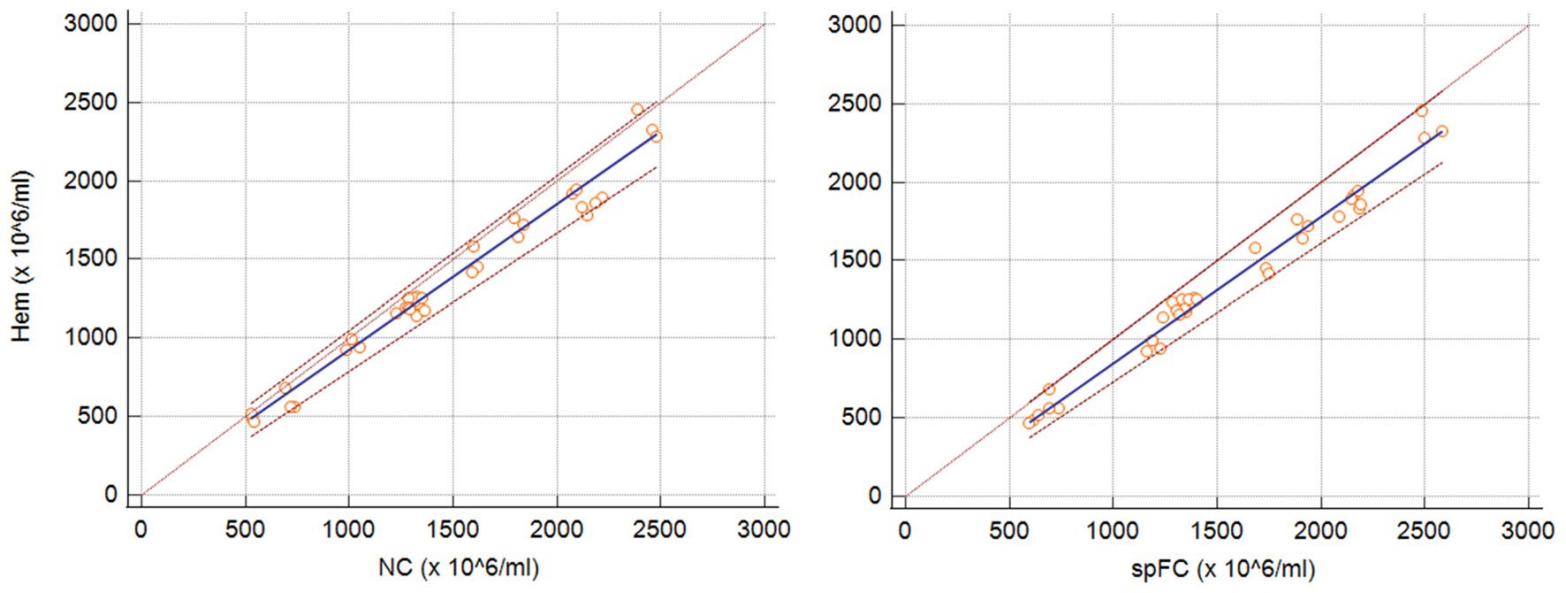
Passing-Bablok regression graph showing the agreement between concentration measured with haemocytometer (Hem) versus NucleoCounter (NC) on the left, and with Hem versus flow cytometry using fluorospheres (spFC) on the right. The dotted brown line represents the 45°-identity line (x = y), the solid blue line represents the regression line, and the dashed brown lines represent the confidence interval for the regression line.

**Figure 2:**
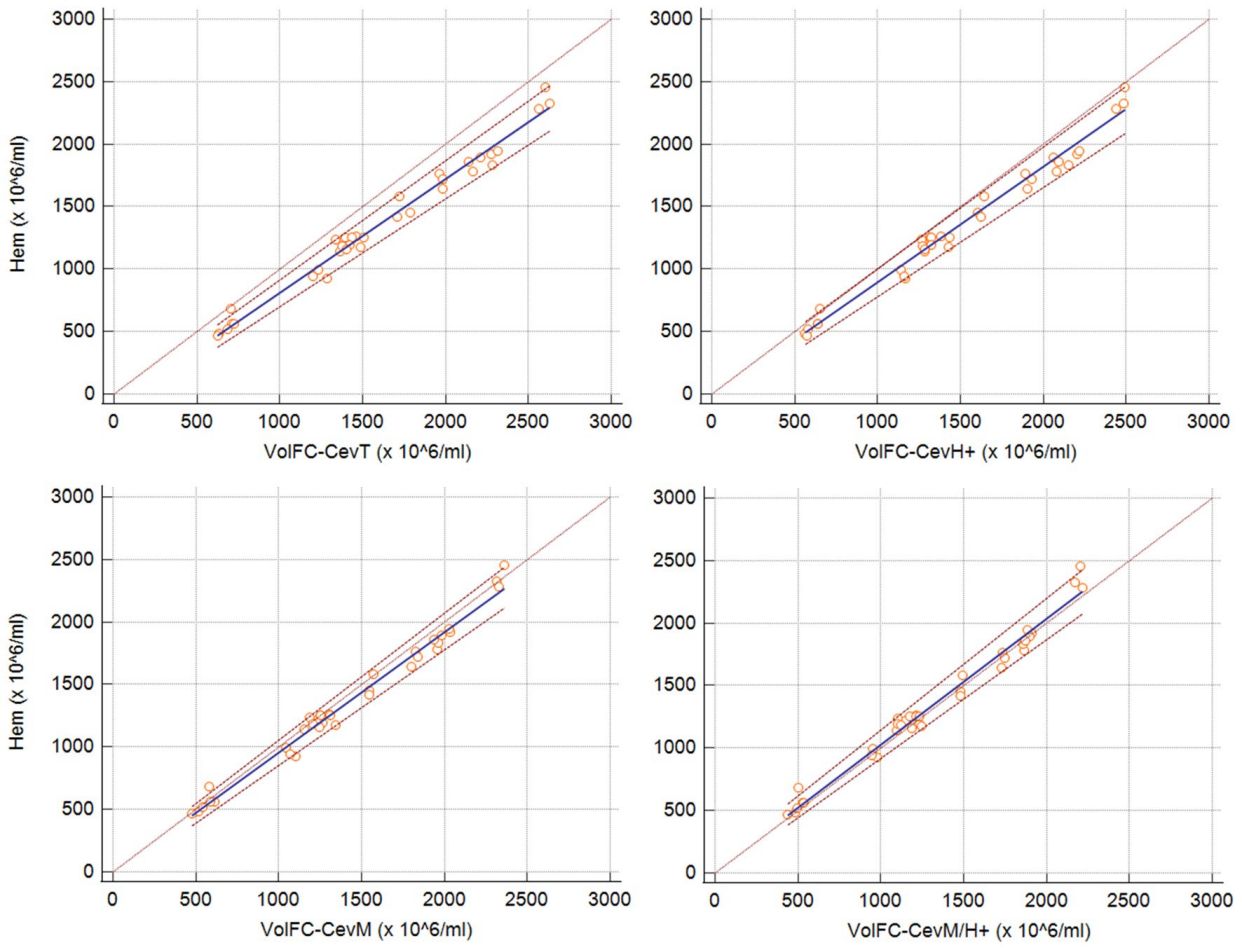
Passing-Bablok regression graph showing the agreement between concentration measured with a haemocytometer (Hem) versus total events estimated with volumetric flow cytometry (volFC-CevT), events positive to Hoechst 33,342 (volFC-CevH +), events gated on sperm morphological criteria (volFC-CevM), and events gated on both sperm morphology and Hoechst positivity (volFC-CevM/H +). The dotted brown line represents the 45°-identity line (x = y), the solid blue line represents the regression line, and the dashed brown lines represent the confidence interval for the regression line. Note that Hem vs volFC-CevM/H + overlap closely with the 45 °-identity line, and Hem vs volFC-CevT is more divergent.

**Figure 3:**
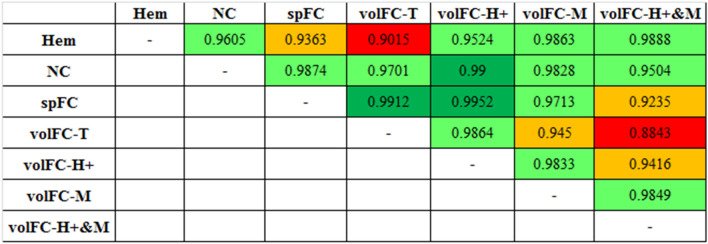
Agreement strength, quantified with Lin’s concordance correlation coefficient (CCC), between the different techniques used to estimate concentration. According to^39^, CCC > 0.99 was considered almost perfect (dark green), CCC between 0.95 to 0.99 was defined as substantial (light green), CCC between 0.90 to 0.95 was considered moderate (orange), and CCC < 0.90 was poor (red).

The agreement between Hem and NC and spFC was substantial (ρ_c_ = 0.9605) and moderate (ρ_c_ = 0.9363), respectively (Fig. 1). A variable agreement was recorded for Hem and the different populations of the volFC, with a substantial agreement with CevM/H + (ρ_c_ = 0.9888), CevM (ρ_c_ = 0.9863), and CevH + (ρ_c_ = 0.9524), while only moderate for CevT (ρ_c_ = 0.9015) (Fig. 2). Lin’s CCC for the agreement between the different techniques is shown in Fig. 3.

### Trial 4. Evaluation of cryopreserved sperm concentration with volumetric flow cytometry and NucleoCounter

**Figure 4:**
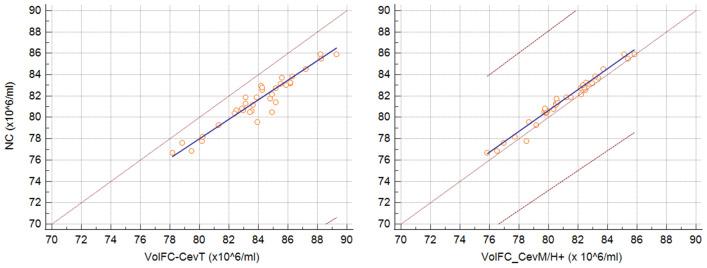
Passing-Bablok regression graph showing the agreement between concentration measured with NucleoCounter (NC) versus total events estimated with volumetric flow cytometry (volFC-CevT), on the left, and NC versus events gated on both sperm morphology and Hoechst positivity (volFC-CevM/H +) on the right. The dotted brown line represents the 45°-identity line (x = y), the solid blue line represents the regression line, and the dashed brown lines represent the confidence interval for the regression line. Note that NC vs volFC-CevM/H + overlap closely with the 45 °-identity line, while NV vs volFC-CevT is more divergent and the dashed lines of the confidence interval are not visible, because out of the graph area.

The cryopreserved samples showed less differences between concentrations estimated with volFC and NC. The mean concentration of the cryopreserved insemination doses was 80 ± 2.48 × 10^6^/ml for NC, 83.70 ± 2.66 × 10^6^/ml for volFC-CevT, 80.04 ± 2.35 × 10^6^/ml for volFC-CevM, 81.71 ± 2.43 × 10^6^/ml for volFC-CevH + , and 78.6 ± 2.56 × 10^6^/ml for volFC-CevM/H + , with no significant differences (*P* > 0.05). The NC showed an almost perfect agreement with volFC-CevM (ρ_c_ = 0.9898), and substantial with volFC-CevH + (ρ_c_ = 0.9525, Fig. 4) and volFC-CevM/H + (ρ_c_ = 0.9621). On the other hand, volFC-CevT showed poor agreement with NC (ρ_c_ = 0.6354; Fig. 4) and all the other volFC populations (volFC-CevH + , ρ_c_ = 0.7565; volFC-CevM, ρ_c_ = 0.6593; volFC-CevM/H + , ρ_c_ = 0.5384).

## Discussion

Although some authors hypothesized the total number of spermatozoa in the ejaculate is the proper parameter to estimate the spermatogenetic efficiency^1^, the precise estimation of sperm concentration, as the cells in the unit of volume, is a stronghold for both clinical and technological implications^12,21^. Whatever the parameter considered, namely total sperm in the ejaculate or sperm concentration, the accurate count of spermatozoa in a known volume is crucial to achieve a reliable estimation of male function. Assuming that no technique guarantees the exact count of the spermatozoa in a specific volume, an efficient analysis allows a better approximation of the real estimation.

The volFC was found solid in the estimation of the sampling volume. The preliminary trials, using fluorospheres at certified concentration, showed that repeated measures on the same sample resulted in consistent results, with negligible effects of the variables tested, namely the dilution or the worn-out.

A previous study on human spermatozoa implemented a new flow cytometer in the assessment of sperm concentration using a volumetric module^20^, although the technology at the basis was not reported. Recently, a technology similar to that used in the present study (clinical version) was validated for an absolute count of CD4 + lymphocytes in human blood samples^19^.In both studies, the stability of the sample volume of the device was not tested.

Among the different techniques, the use of NucleoCounter became popular because of its high precision and accuracy coupled with rapidity and simplicity^12^. In a multicentric study, the same samples analyzed by the NC showed an intra-laboratory coefficient of variation between 2.7 and 6.8%. Consistently with these previous studies, here the variation between replicates using the NC was 1.8% in fresh samples and 0.63% in cryopreserved samples, near the lower limit of the range previously described^22^. Interestingly, trial 1 showed that the key factor during the estimation of concentration with NC was the dilution of the sample. Increasing dilution rate resulted in a progressive increase in the concentration measured. The large dilution required by the ejaculate concentration in the bull together with the small volume read by the device^15^, approximately 1 µl of diluted sample^12^, could explain this effect. This finding underlines the importance to choose correctly the proper dilution factor, following closely the Manufacturer’s instructions. At the proper dilution rate, the concentration appeared consistent with the other methods. This hypothesis is corroborated by the relevant reduction in the CV recorded in cryopreserved samples, in which the concentration is lower compared with fresh ones.

Differently from the NC, volFC appeared reliable and stable in the results in a large range of dilution (between 1:200 and 1:800). The stability of the results at different dilution rates confirmed other studies on humans^20^ and rats^23^ in which the FC was effective also with high diluted samples. Greater attention, however, should be devoted to selecting the correct population of interest for this analysis. The findings showed that the total number of events could be misleading since a relevant subpopulation of events with morphological criteria comparable with spermatozoa but without stained DNA was detected. These results corroborated the hypothesis of a previous study, in which a systematic overestimation of spermatozoa in non-stained populations due to other non-cellular debris within the sperm morphological gate was mathematically demonstrated^24^. On the other hand, the definition of sperm concentration based only on the DNA content could also overestimate the real value. In the present study, a discrepancy between the size of the Hoechst positive subpopulation and the sperm morphology/Hoechst positive subpopulation suggested that a proportion of Hoechst positive elements are not spermatozoa. Previous studies showed that fresh semen contains a variable degree of bacterial contamination from the genital tract^25–28^. Furthermore, other non-sperm cells, including white blood cells and epithelial cells, are present in the semen of mammals and can be detected flow cytometrically^29–32^. In both these cases, the location of these cells on the FCS-SSC plot was out of the usual morphological region of spermatozoa. For this reason, in the present study, the estimation of sperm concentration via volFC was tested also on the subpopulation selected with the double morphological and DNA positivity gate. The rightness of this approach was demonstrated by the better agreement of this latter subpopulation with the haemocytometer, in which only visualized spermatozoa were considered.

Similarly, to avoid the misleading effect of the non-sperm cells or debris in the sample, also the sperm concentration was estimated flow-cytometrically using the fluorospheres focused on fluorescent gates, assuming that these cells were spermatozoa^12,17^. Similar conclusions were reached by the authors of a previous study on humans in which the concentration estimated using the spFC was significantly improved when the combination of morphological and DNA fluorescent gates was applied to the semen analysis via flow cytometry^21^.

Among the different techniques considered in the present study to estimate the concentration, the haemocytometer showed a larger variability. It is generally admitted that the intra-observer variability with Hem is near 10%^10,33^, but with standardized procedures and trained technicians, the variability could be reduced to less than 5%^21^. Thus, it is not surprising that, with the standardized procedure performed by a trained operator, the variability recorded in the bovine semen was 4.52%. On the other hand, the evaluation with haemocytometers, although it is the gold standard, is subjective and time-consuming. The automated methodologies proposed in the present study showed lower variability. NucleoCounter coefficient of variation was consistent with previously reported intra-laboratory CV^13,14,22^, confirming the good repeatability performance of this device. The flow cytometry, in both the indirect (calculated on the basis of fluorospheres at known concentration) and volumetric method, confirmed the effectiveness of this device in performing replicable analysis. In the present study, the spFC showed a CV of 2.14%, consistent with other studies based on the same technology^10,14,21^. Similarly, the CV in volFC was between 1.97 and 1.94% in CevM and CevT, respectively, and 1.24% in CevH+. The larger CV recorded in subjective and manual procedures, such as the haemocytometer, compared with the automated methods, demonstrated in several studies^20,21^ could be attributable to the number of elements (a few hundred for Hem, tens of thousands for the most automated methods) used to estimate the concentration, suggesting these last ones are more representative of the whole sample.

Cryopreservation does not affect the results obtained by volFC. The presence of debris and diluted components could affect the effectiveness of several methods. Some methodologies, such as spectrophotometers, could not be used on this matrix, due to the presence of glycerol^15^. On the other hand, NC and volFC on stained spermatozoa appeared relatively specific since in both cases the DNA of the cell was used for event detection, reducing the interference by extender components and debris^15^. The findings suggested that the volFC showed consistent results compared with NC, especially in cryopreserved samples, but is less prone to the effect of the dilution rate and, in turn, to the concentration of the raw semen.

## Conclusions

Flow cytometry implemented with volumetric technology is a accurate and precise method to estimate sperm concentration in bovine fresh and frozen semen, avoiding the potential bias arising from the use of fluorospheres or the dilution rate. Although currenty this device has higher costs and requires skilled operators, the progressive diffusion of simplified flow cytometers with standardize kits for semen analysis favour the spread of this technology in field conditions^34^. The validation of the volFC could lay the basis for the improvement in the quantification of subpopulations with specific functional, metabolic, or structural properties, implementing the possibility to explore the relationships between sperm attributes and fertility.

## Methods

The trials reported in the present manuscript received the approval by the Ethical Committee of the Department of Veterinary Medicine, University of Teramo-Italy (protocol n. 16,571—09/06/2023).

### Preliminary procedures

In this study, volume-variable micropipettes (model Acura 825, volume range: 0.5–10 μL; 10–100 μL; 100–1000 μL. Socorex Isba SA, Ecuclens, Swiss) were used to perform dilution of the samples. All the micropipettes used in this study were validated gravimetrically before the experiments for the specific volume they displaced. The medium used for the gravimetric validation was bi-distilled water, prepared a few minutes before the test. For each micropipette, the volume to check was dispensed on a certified analytic balance (Explorer, Ohaus Corp., Parsippany, NJ, USA). The precision, as the degree of closeness between repeated measurements of the same volume, was calculated on 20 measures in triplicate, by the calculation of the coefficient of variation (CV) and the intraclass correlation coefficient (ICC-see the statistical evaluation section for more details).

The threshold for acceptable precision was 2% of CV and R = 0.98 for ICC: values beyond the thresholds required calibration. In this study, no micropipette was found beyond the threshold set-up.

The standardization of the procedures of dilution was increased as much as possible. Briefly, the tip was systematically dried externally to remove any external micro drops. After dilution, the sample was mixed using a different micropipette able to displace 90% of the final volume, pipetting several times (20 folds) and avoiding bubble formation. In this study, vortex mixing was not performed, since seminal plasma and media created bubble formation in the sample.

The flow cytometric analyses were performed using the CytoFLEX (Beckman Coulter Inc., Brea, CA, USA) equipped with three lasers (wavelength of 405 nm–80 mW; 488 nm–50 mW; 638 nm–50 mW). Data were managed by the CytExpert software version 2.1 (Beckman Coulter). For all the trials, volumetric flow cytometric (volFC) analyses were performed at a flow rate standardized at 10 μL/min, and the analysis was stopped using the volumetric limit of 20 μL. Events were recorded systematically 30 s after the beginning of the analysis, to allow the stabilization of the fluidics.

### Preliminary trial

The VolFC analysis was preliminary verified using standard Flow-Count fluorospheres (Batch 7548247F) at certificated concentration, estimated by the manufacturer in 1014 beads/μL (for the batch used in this study). In details, fluorospheres were diluted 1:2, 1:4, 1:8, and 1:16 (v:v) with bi-distilled water. A sample was not diluted and raw-analysed. After an appropriate resuspension, performed as above described, the samples were analysed using the flow cytometer CytoFLEX (Beckman Coulter). Samples with progressive fluorosphere dilution were prepared 3 times, as replicates. Total events (beadsT), and events excited with 488 nm and recorded with the 585/42 band-pass filter (beads488), and the original concentration of beads was calculated with the following formula:

$$Concentration = number \, of \, events \, * \, 50 \, * \, dilution$$

Thus, total beads concentration (CbeadsT) and beads concentration at 488 nm (Cbeads488) were calculated.

To verify the precision of fluorosphere concentration, 6 aliquots of the same batch were undiluted (1:0) or diluted (1:4) and analysed in 6 replicates.

To estimate if the worn-out fluidic components could affect concentration estimation, 6 samples of diluted beads (1:4, v:v) were compared using a worn-out (serial number 20192241; approximately 600 working hours) or new peristaltic pump tube (serial number 20210758).

### Animals and semen collection

A total of 12 mature bulls were included in these trials. The bulls (2–7 years old) were housed in the Alpenseme Artificial Insemination Centre of the Provincial Breeders Federation of Trento (Ton, Trento, Italy). All the animals were routinely collected twice a week within the artificial insemination program and aliquots of the ejaculates were removed to carry out the trials. For this reason, no semen collections were performed specifically for this study. The animals were managed according to the European Commission Directive for Farm Animal Welfare (Directive 98/58/EC) and the National Law for Animal Welfare and Protection (Italy). Collections were performed by the same operator using a pre-warmed artificial vagina.

After collection, an aliquot was removed and used for the experiments (fresh samples-trials 1–3). Then, the semen was processed to produce cryopreserved artificial insemination doses, part of which was used for the procedures on cryopreserved samples (trial 4). Briefly, volume was estimated by weight using a precision balance CP6201 (Sartorius AG, Gottingen, Germany), assuming 1 mL = 1 g^35^. Concentration was evaluated photometrically using bovine-specific Accucell (IMV Technologies, L’Aigle, France) after dilution 1:100 with saline solution^26^. Semen was diluted at approximately 100 × 10^6^ sperm/mL with Bioxcell (IMV Technologies) and equilibrated for 3 h at 5 °C in a passive refrigerator. After equilibration, diluted semen was packaged in 0.25-mL straws, frozen with a programmable nitrogen freezer (Digicool 5300, IMV Technologies), with the following cooling rate (− 5 °C/min from + 4 to − 10 °C; − 40 °C/min from − 10 to − 100 °C; and − 20 °C/min from − 100 to − 140 °C)^36^, and plugged in liquid nitrogen for storage. Seven days after freezing, 8 straws/ejaculate were thawed in the water bath at 38 °C for 1 min and evaluated for concentration (trial 4).

### Trial 1. Effect of dilution on concentration estimated with a volumetric flow cytometer and NucleoCounter

To verify the effect of the sample dilution on the sample concentration, progressive dilutions (1:12.5; 1:25; 1:50; 1:100; 1:200; 1:400; 1:800) of the sample were prepared and analysed using the volFC and NC SP-100 (ChemoMetec, Allerod, Denmark). The micropipettes used for sampling were the same between methods. Samples for volFC were diluted with phosphate-buffered saline (PBS) sterilized by filtration (Filtropur S, pore size 0.2 μm, Sarstedt AG & Co, Nümbrecht, Germany) added with Hoechst 33,342 (ENZ-52401, Enzo Life Sciences AG, Lausen, Switzerland) at the final concentration of 1.69 μM (PBS-H).

The volFC samples were incubated for 15 min at 37 °C^37^ and analysed using the CytoFLEX (Beckman Coulter) using the 405 nm laser (excitation). Events were detected using the 450/45 band-pass filter. The flow rate was standardized at 10 μL/min, and the analysis was stopped using the volumetric limit of 20 μL. Events were recorded systematically 30 s after the beginning of the analysis, to allow the stabilization of the fluidics. A morphological gate was created on the forward scatter x side scatter plot to detect only sperm-referred events. A 450-fluorescent gate was created on the event histogram, to detect only events positive for the Hoechst 33,342 (H+). Finally, a double morphological and fluorescent gate was created. Total events (evT), morphological gated events (evM), fluorescent gated events (evH+), and events selected by morphologically and fluorescence (evM/H+) were recorded. The original concentration of the sample was calculated with the following formula:

$$Concentration \, = \, number \, of \, events \, * \, 50 \, * \, dilution$$

Samples for NC were diluted with SP-100 diluent (ChemoMetec). Before the analysis, the sample was systematically mixed using a different micropipette able to displace 90% of the final volume, pipetting several times (20 folds) and avoiding bubbles formation. Then, the resuspended sample was loaded in SP1-cassette with a waiting period standardized at 10 s before the analysis, inserted in the device taking care to adjust the dilution factor, and analysed. The concentration that appeared on the display, was recorded and used for the statistical analysis. The prevalence of the error messages provided by the NucleoCounter (Error: Sample could not be analysed) at the different dilutions was also recorded.

### Trial 2. The repeatability of volumetric flow cytometry compared with NucleoCounter to estimate sperm concentration

Aliquots of fresh semen from the 12 bulls were pooled (4 bulls/pool) to create three pooled samples. Each pool was then examined 10 times (replicates) using both the volFC and NC, to estimate the repeatability of both methods. For the volFC, samples were prepared at dilution 1:400 with sterilized PBS-H and incubated for 15 min at 37 °C and analyzed flow-cytometrically as described above. Total events (evT), morphological gated events (evM), fluorescent gated events (evH+), and events selected by morphologically and fluorescence (evM/H+) were recorded. The original concentration of each category in the sample was calculated with the following formula:

$$Concentration = number \, of \, events \, * \, 50 \, * \, 400$$

For the NC, repeated analyses were performed on the same samples after dilution 1:401 using the SP-100 diluent (ChemoMetec), following the Manufacturer’s instruction, selecting bulls as to the species and 401 as to the dilution factor. In brief, 25 μL of fresh semen was transferred in a sterile cup, and 10 mL of SP-100 was dispensed (Dispensette III bottle-top dispenser, Brand, Wertheim, Germany) in the cap. Then, SP1-cassettes were prepared as described above and analysed.

### Trial 3. Comparison of different methods to estimate the concentration

Fresh samples from each of the 12 bulls were prepared in triplicate and analysed with different methods to estimate concentration: (1) haemocytometer (Hem); (2) NucleoCounter; (3) flow cytometry with fluorospheres; (4) volumetric flow cytometry.

Evaluation of concentration by Hem was performed using a Bürker counting chamber (Merck, Leuven, Belgium) after dilution 1:1000 with 0.9% NaCl solution with 3% glutaraldehyde to ensure sperm immobilization. At least 400 spermatozoa in two chambers were counted to estimate concentration. If the difference of spermatozoa in each chamber exceeded 10% compared to the other of the same slide, the sample was re-prepared. Each sample was prepared in triplicate.

The NC was used as recommended by the manufacturer’s instructions. The bull was selected as species, and the dilution factor inserted was 401. Samples were prepared as above mentioned. After loading in the SP1-cassette, a waiting period of 10 s was observed, and then the sample was analysed. Each sample was prepared and analysed in triplicate. The concentration reported on the display was recorded and used for statistical analysis.

The estimation of sperm concentration by spFC was performed using Flow-Count Fluorospheres (Beckman Coulter) at certified concentration, using the batch above mentioned. The sample was diluted 1:200 using sterilized and stained PBS (Hoechst 33,342, dilution 1:1000 with PBS-H). Then, the sample was further diluted 1:1 (v:v) with fluorospheres and analyzed with CytoFLEX (Beckman Coulter). Each sample was prepared in triplicate. Events excited at 405 nm and recorded at 450/45 nm (spermatozoa) and events excited at 488 nm and recorded at 585/42 nm (fluorospheres) were used to estimate sperm concentration using the following formula:

$$spFC \, concentration = \left[ {\left( {\left( {405 - events*200} \right)/\left( {488 - events} \right)} \right)*fluorospheres \, concentration} \right]$$

The analysis, performed at the flow rate of 10 µl/min, was stopped at 20,000 events.

For the volFC, concentration was calculated after dilution of the semen at 1:400 with sterilized PBS-H. Samples were incubated for 15 min at 37 °C, mixed as above mentioned soon before the analysis, performed with the CytoFLEX flow cytometer (Beckman Coulter). Each sample was prepared in triplicate. The flow rate was standardized at 10 μL/min, and the analysis was stopped using the volumetric limit of 20 μL. Events were recorded systematically 30 s after the beginning of the analysis, to allow the stabilization of the fluidics. A morphological gate, 405-fluorescent gate, and double morphological plus fluorescent gate were created and events in the respective gate were recorded as total events (evT), morphological gated events (evM), fluorescent gated events (evH+), and events selected by both morphological and fluorescent gate (evM/H+). The original concentration of the sample was calculated with the following formula:

$$Concentration = number \, of \, events \, * \, 50 \, * \, 400$$

### Trial 4. Evaluation of cryopreserved sperm concentration with volumetric flow cytometry and NucleoCounter

A total of 8 straws for each bull included in the present study were thawed in a waterbath (38 °C) for 60 s, then samples were transferred in a sterile 5-ml tube. For volFC, samples were diluted 1:40 with sterilized PBS-H and incubated for 15 min at 37 °C. After proper resuspension, the sample was analysed flow-cytometrically as described above. Each sample was prepared, for the analysis, in triplicate. Total events (evT), morphological gated events (evM), fluorescent gated events (evH+), and events selected by morphologically and fluorescence (evM/H+) were recorded and used to calculate the original concentration, using the formula:

$$Concentration = number \, of \, events \, * \, 50 \, * \, 40$$

For the NC, samples were diluted 1:51 using the SP-100 diluent (ChemoMetec) in a sample cup, according to the Manufacturer’s instruction. The dilution factor of 51 was set on the device. Then, SP1-cassettes were prepared as described above. The dilution of each sample was performed in triplicate.

### Statistical analysis

Data were presented as mean ± standard deviation (SD). The normal distribution of the data was tested using the Shapiro–Wilk test. Furthermore, homoscedasticity between groups was tested by Levene’s test. Data in the present study were normally and homogeneously distributed.

#### Preliminary trial

Differences in the recalculated concentration between different dilutions were performed using a general linear model (GLM) based on ANOVA, in which the dilution factor was considered a fixed variable. A Scheffè *post-hoc* test was performed when appropriate. The proximity between the concentration calculated at the different dilutions and the certified concentration of the fluorospheres was calculated as a percentage value (100% = certified concentration), and the GLM was used to verify the effect of the dilution, followed by the Scheffè test for the *post-hoc* analysis. The repeatability of the analysis in the undiluted (1:0) and diluted samples (1:4) was estimated by the ICC^38^. Finally, the effect of worn-out fluidic components on concentration estimation was checked by the t-Student test for paired values. In all the cases, significance was set at *P* < 0.05.

#### Trial 1

In this trial, the concentration of spermatozoa at increasing dilution was estimated with volFC for each sample using the following flow cytometric populations: evT, evM, evH + , and evM/H + . Differences between concentrations in the different populations of the volFC and NucloCounter, at correspondent dilution (1:12.5–1:800), were tested using the GLM. Dilution and concentration with the different techniques (NC, CevT, CevM, CevH+, and CevM/H+) were considered fixed factors. The general linear model was followed by the post hoc Scheffè test when appropriate. Significant differences were considered with *P* < 0.05.

#### Trial 2

The repeatability of concentration was measured with volFC, using the different populations (evT; evM; evH+; evM/H+), and NucleoCounter was estimated in three pools of bovine fresh semen by calculating the ICC on 10 replicates, as above mentioned^38^.

#### Trial 3

Differences in the concentration estimated with the different techniques, namely Hem, NC, spFC, and volFC (calculated on the different populations-evT; evM; evH+; evM/H+) were tested using a general linear model (GLM) based on ANOVA. The reproducibility was quantified by the variation between replicates, using the coefficient of variation^21^. The agreement between the different techniques was calculated by Lin’s concordance correlation coefficient (CCC-ρ_c_) with a strength of agreement as follows: > 0.99, almost perfect; between 0.95 and 0.99, substantial; between 0.90 and 0.95, moderate; < 0.90, poor^39^. Graphical agreement between techniques was performed using the Passing-Bablok regression plot^40^.

#### Trial 4

The concentrations of cryopreserved semen from the 12 bulls included in the study estimated using volumetric flow cytometry (different populations) and NucleoCounter were compared. Differences between concentrations in the different populations of the volFC and NucloCounter were tested using the GLM. The different techniques or populations considered (NC, CevT, CevM, CevH+, and CevM/H+) were considered fixed factors. The general linear model was followed by the post hoc Scheffè test. Significant differences were considered with *P* < 0.05. The agreement between the different techniques to estimate sperm cryopreserved concentration was tested by calculating Lin’s CCC. Graphical agreement between techniques was performed using the Passing-Bablok regression plot^40^.

### Supplementary Information

Supplementary Information.

## Data Availability

The datasets used and analyzed during the current study are reported in the supplementary material. Additional raw data files are available from the corresponding authors upon reasonable request.
